# New graduate nurses’ experiences with and perceptions of their mental health and well-being during the COVID-19 pandemic: An interpretive description study protocol

**DOI:** 10.1371/journal.pone.0315852

**Published:** 2025-02-28

**Authors:** Robin D. Burry, April Pike, Joy Maddigan, Peggy Rauman, Holly Burford, Joanne Smith-Young, Vernon Curran

**Affiliations:** 1 Faculty of Nursing, Memorial University of Newfoundland, St. John’s, Newfoundland and Labrador, Canada; 2 Faculty of Medicine, Memorial University of Newfoundland, St. John’s, Newfoundland and Labrador, Canada; Public Library of Science, UNITED KINGDOM OF GREAT BRITAIN AND NORTHERN IRELAND

## Abstract

**Background:**

The COVID-19 pandemic has had a significant impact on healthcare workers. Healthcare workplaces are high stress environments placing care providers such as Registered Nurses at high risk for occupational stress injuries related to poor mental health. Currently, healthcare authorities rely on new graduate nurses to help fill gaps in staffing; however, novice nurses are especially vulnerable to workplace illnesses, with the recent pandemic contributing to this risk. Research is needed to understand new graduate nurses’ experiences and perceptions of their mental health and well-being as they transition to practice in the COVID-19 pandemic and the supportive resources they require to assist in contributing to healthy workplaces.

**Objectives:**

1) To explore new graduate nurses’ experiences and perceptions of their mental health and well-being as they transitioned to practice during the COVID-19 pandemic in Newfoundland and Labrador, Canada; 2) To understand new graduate nurses’ awareness and use of available mental health supports and resources during the COVID-19 pandemic; and 3) To identify strategies and resources to support new graduate nurses’ mental health and well-being as they transitioned to practice during a public health crisis.

**Methods:**

An interpretive description research methodology will be used to conduct this study. The COnsolidated criteria for REporting Qualitative research (COREQ) checklist will be used to verify both the structure of the study and presentation of findings. Approximately forty semi-structured interviews will be conducted with new graduate nurses who worked in Newfoundland and Labrador in 2020, 2021 or 2022. Data collected will be analyzed using thematic analysis with descriptive statistics used to present demographic information.

**Results:**

The results of this study will help inform changes to existing workplace programs and contribute to the development of new processes to support new graduate nurses’ mental health and well-being as they transition to practice during a public health crisis such as COVID-19.

## Introduction

The Severe Acute Respiratory Syndrome Coronavirus 2 ([SARS-CoV-2] COVID-19) pandemic has had a significant impact on healthcare systems around the world and on the essential workers who provide care. Mental health is an important aspect to be considered when evaluating the health and safety of workers and workplaces. The effects of the COVID-19 pandemic on the mental health and well-being of healthcare workers (HCWs) have only begun to be understood [1,2]. Unmanaged mental health challenges in the workplace can have a negative impact on the overall safety of workers and workplaces including: absenteeism, attrition, and patient safety issues [3–6]. Of concern is the mental health and well-being of new graduate nurses (NGNs); Registered Nurses (RNs) who have graduated from an accredited nursing program, passed the nursing registration exam, received the protected title of Registered Nurse (RN), and are in their first two years of nursing practice.

New graduate nurses are known to face specific challenges during their first years of practice and are therefore particularly vulnerable when the added stressors of a pandemic are taken into consideration [7–10]. With the healthcare workforce under unprecedented pressure during the COVID-19 pandemic, NGNs were called upon to fill gaps in staffing and played an important role in the provision of care. Protecting those providing care in a public health crisis is a key factor in safe-guarding individual patients and the community at large. Further research is needed to understand the experiences of NGNs as they transitioned to practice during the COVID-19 pandemic and the supportive resources that they require to build and maintain their mental health and well-being. This research will not only help to inform current practices, but will assist in preparing for future public healthcare crises.

The goal of this study is to understand NGNs’ experiences with and perceptions of their mental health and well-being as they transitioned to practice during the COVID-19 pandemic in Newfoundland and Labrador (NL). This will help researchers to understand the nature and type of support resources accessed and used, as well as identify strategies and resources to support NGNs’ mental health and well-being as they transition into practice and during a public health crisis. Interviews will be conducted with NGNs in their first two years of practice working in NL.

## Literature review

Health and safety are important components upon which our healthcare system is based. However, due to pre-existing healthcare worker (HCW) staffing shortages, and additional pressures brought on by the advent of the COVID-19 pandemic, the health and safety of healthcare workplaces in NL is in question [11]. NGNs are particularly vulnerable as they are still learning amidst the challenges of COVID-19. NGNs’ experiences and perceptions of their mental health and well-being as they transitioned to practice during the COVID-19 pandemic in NL is currently unknown as are the supportive resources they may have required or utilized.

The COVID-19 pandemic has had a substantial impact on all facets of life around the world. While public health measures such as lockdowns were instituted, HCWs continued to work to support the health and well-being of individual patients and communities. RNs played a vital role in protecting the public and caring for those who were ill throughout the pandemic; however, this role did not come without a price. Although media reports of the negative impact of the COVID-19 pandemic on HCWs are widespread, the physical, psychological and social health impact of RNs has only begun to be studied and understood [1,2]. High workloads due to lack of staffing, availability and knowledge of personal protective equipment (PPE), and fear of infection and transmission during the pandemic are just a few of the added stressors contributing to the mental health burden carried by nurses [12,13]. Continued exposure to these types of stressors can result in mental health occupational stress injuries such as secondary and posttraumatic stress disorders, depression, anxiety, and burnout [14–19]. These injuries can exacerbate staffing shortages and negatively impact patient care and safety [3–6].

Worldwide staffing shortages in healthcare were predicted well before the arrival of the COVID-19 pandemic with the World Health Organization (2020) having forecasted a worldwide shortage of 4.6 million nurses by 2030 [20] and the Canadian Nurses Association (2009) predicting a shortfall of 60,000 RNs by 2022 [21]. The predicted staffing shortage before the pandemic has exponentially increased as a result of the current public health crisis caused by COVID-19. Globally, 80% of nurses in most countries are reporting mental distress and a predicted attrition rate of up to 15% is projected equating to a projected shortfall of 14 million nurses by 2030 due to the “mass traumatization” of nurses by the COVID-19 pandemic [11]. We have already begun to see the effects of this COVID-19 induced trauma with RNs resigning or retiring citing poor work conditions, staffing shortages and mental fatigue or burnout [22].

Nurses’ experiences in previous pandemics such as Severe Acute Respiratory Syndrome (SARS), Middle East Respiratory Syndrome Coronavirus (MERS-CoV), Avian influenza A virus (H5N1) and novel influenza A subtype ([swine flu] H1N1) were reported in a recent systematic review and found staffing shortages played a major role in how nurses coped with work demands [23]. In a qualitative descriptive study conducted with 24 nurses working in sub-intensive care units during the COVID-19 pandemic, nurses were often reassigned to unfamiliar units to assist with staffing challenges resulting in a negative impact on nurses’ mental well-being with feelings such as fear, uncertainty, inadequacy and unpreparedness [24]. A recent cross-sectional study of 1005 nurses working in Italian hospitals, reported that the pandemic resulted in nurses’ experiencing sleep disturbances (71.4%), moderate anxiety (33.23%) and low self-efficacy (50.65%) with a positive correlation found between anxiety and sleep quality (0.408; *p* < .0001) [25]. Furthermore, results of a retrospective cohort study conducted in the United States of suicides from 2007 to 2018 found a higher rate of suicide in women nurses when compared to women in the general population [26,27]. Disturbingly, the rate of suicide in women nurses is speculated to have increased as a result of the COVID-19 pandemic [26,27].

Proposed solutions to the nursing staffing shortage before and during COVID-19 frequently involved the recruitment and retention of NGNs, as they are the largest available cadre of nurses from which to recruit [28]. However, HCWs who are new to their respective professions, such as NGNs, are challenged with learning while working as they transition from student to independent practitioners. This makes this cohort particularly vulnerable to workplace illnesses such as occupational stress injuries [2].

The transition from student to practicing nurse is also a difficult experience for many NGNs who face multiple challenges in the first years of practice [7–9]. Managing the transition to an unfamiliar work environment with high patient acuity and workloads are just a few of the stressors with which NGNs must cope as they enter professional practice [8–10,29]. Stress, anxiety, self-doubt and frustration are often cited by new nurses during this stage of their careers [27,29], with 26-57% of new graduates leaving their first positions or the profession entirely [30]. This high rate of turnover can have a negative impact on mentors who are repeatedly asked to support new graduate hires contributing to the burnout of experienced nurses [31–33]. These pressures have only increased with novice nurses required to simultaneously manage the stressors related to transition to practice and those presented by the COVID-19 pandemic [1]. In a survey study conducted during the current pandemic, 83 nurses were surveyed with results indicating nurses presented a high risk for depression, anxiety, hyperarousal and burnout, with more novice nurses presenting a higher risk than those with more experience [2]. Of concern was that supportive resources such as mentoring programs were known to be deprioritized as a result of staffing pressures and patient care needs during the COVID-19 pandemic [34]. However, studies have shown these programs to be successful in positively influencing socialization and retention of NGNs [35], with a recent scoping review recommending these types of programs be restructured to enable their continuance during times of crisis to promote a sustainable nursing workforce [36]. Considering novice nurses are being sought to address the worldwide nursing shortage and play a vital role in the provision of care, their transition to practice experience and mental health and well-being are of paramount concern.

Emerging research focused on nurses’ mental health and well-being during the COVID-19 pandemic and NGN transition has been presented; however, a gap in knowledge remains regarding new nursing graduates’ mental health and well-being experiences and perceptions during transition to practice in the COVID-19 pandemic and the supportive resources they require to be retained to support healthy workplaces in NL.

The overarching question guiding this study is: How do new graduate nurses experience and perceive their mental health and well-being as they transition to practice during the COVID-19 pandemic in Newfoundland and Labrador? The three main research objectives are: 1) To explore NGN’s experiences and perceptions of their mental health and well-being as they transitioned to practice during the COVID-19 pandemic; 2) To understand NGN’s awareness and use of supportive resources during the COVID-19 pandemic; and 3) To identify strategies and resources to support NGNs’ mental health and well-being as they transitioned to practice during a public health crisis.

## Materials and methods

A qualitative interpretive description research methodology will be used to conduct this study.^37^ This methodology was determined to be the most appropriate as the focus of this study is to develop an in-depth description of a phenomenon that is challenging to remove from the context, specifically the mental health and well-being of new nursing graduates transitioning to practice during the COVID-19 pandemic [37,38]. A key component of interpretive description is the real world application of study findings [37]. As one of the main objectives of this study to identify strategies and resources to support NGNs’ mental health and wellbeing as they transitioned to practice during a public health crisis, interpretive description is a fitting methodology. Additionally, the COnsolidated criteria for REporting Qualitative research checklist (COREQ) [39] will be used to verify the structure of this study and reporting of findings.

Semi-structured interviews will be conducted using a purposeful, maximum variation approach to sampling [40,41]. This study will be conducted in the five regional zones that make up the Provincial Health Authority (NL Health Services) in Newfoundland and Labrador, Canada and employ NGNs in either in-patient or out-patient settings. This sampling approach is intended to ensure representation from each zone in the province. Data collection will cease when sufficient information has been collected to address the research question and no new meaningful information on the topic of study is revealed with additional interviews [42]. When conducting interviews, Frances et al. (2010) recommends interviewing 10 participants then an additional three [43]. If no new information is disclosed in the three additional interviews, data collection will be considered to be complete. If additional information is revealed, then three more interviews should be conducted with the process continuing until sufficient information has been obtained to address the research question. Although approximately 16 interviews or less are usually sufficient, it is recognized larger sample sizes may be required when studying groups over multiple sites [44]. Therefore, it is anticipated approximately 40 participants will be recruited for this study. Registered nurses who: 1) graduated from a Canadian nursing program between 2020 and 2022; and 2) have worked as a RN in NL will be eligible for this study. Newly registered nurse practitioners and NGNs who only worked as an RN outside of NL will be excluded from this study.

### Recruitment

Responsibility for the recruitment of research participants will rest with the study lead and research assistant (RA). A recruitment email will be sent to eligible participants through the College of Registered Nurses of Newfoundland and Labrador (CRNNL) and Memorial University of Newfoundland, Faculty of Nursing (MUNFON) listserves with two reminder e-mails to be sent at two-week intervals. Information regarding the study will also be shared via email with RNs who fit the inclusion criteria by a representative of the professional practice department of each region. Additional methods will be used to advertise the study and enhance recruitment; for example, study information will be shared via scripted public service announcements on radio stations within the province and on MUNFON’s social media accounts. Registered nurses who are interested in participating in the study will be asked to notify the study lead or RA who will arrange an interview time and method that is convenient for the participant. Recruitment of participants will cease when sufficient information to address the research question is reached [43].

### Data collection and analysis

The study lead or RA will obtain consent from the participants, collect the demographic information and conduct the interviews. Participants who contact the study lead or RA and express interest in participating in the study will be emailed a consent form and study information page to read. At the time of the interview, the interviewer (study lead or RA) will review the study information and consent form and answer any questions from the participant. Verbal consent will be obtained before proceeding with the interview. Demographic information collected will include age, gender, level of education, name of RHA where employed, area of practice, months of experience as a RN, months of experience in current position, and type of nursing position. A one 1-hour semi-structured interview, with the potential for one 15-30 minute follow-up interview, will be conducted with each participant by telephone or online videoconferencing using an interview guide [45]. The interview guide questions [S1_Appendix] will focus on the participants’ descriptions of experiences and perspectives of their mental health and well-being during their transition to practice. Questions regarding supportive resources related to mental health and well-being will also be asked. Interviews will be audio-recorded and transcribed verbatim. Each interview will be de-identified and given a unique code to protect the confidentiality of the participant while maintaining an audit trail. Audio recordings of the interviews will be destroyed once each has been de-identified, coded, transcribed and verified.

Qualitative analysis of the interviews will be conducted using Braun & Clarke’s (2006) [46] approach to thematic analysis which is congruent with the inductive analytical requirements of interpretive description [37,46]. Themes will be identified through Braun & Clarke’s (2006) [46] 6-step process: familiarization with the data, generating initial codes, searching for themes, reviewing themes, defining and naming themes and producing the report. NVIVO qualitative analysis software will be used to track codes and themes. At least two members of the research team will audit results of the analysis. If required, decisions regarding analysis will be reached by consensus of the research team members. Thematic findings from the interviews will be summarized and shared with participants to determine confirmability and transferability [47]. It is important to note that all members of the research team who will be involved in the analysis of the data and interpretation of the results are RNs or specialists in medical education. Interpretive description requires the acknowledgment of the disciplinary lens through which the researcher inherently recognizes “associations, relationships, and patterns within the phenomenon being described” [37, p.56] and enables results to move beyond description to interpretation with potential real world application. Descriptive analysis of demographic data will be analyzed using Microsoft Excel version 2018 and presented in a table.

### Data management

Electronic data will be stored on a password-protected computer. Any hard copy information will be kept in the study lead’s locked cabinet in their locked office at MUNFON. The coding list linking participant names to codes will be stored separately from the study data. Specifically, the coding list will be stored in a separate locked cabinet where no other data pertaining to this study will be stored. A summary of deidentified research data will be made publicly available when the study is completed and published. After five years, paper records will be shredded by local National Association of Information Destruction (NAID) certified shredding facility. Electronic data will be disposed of by ensuring the drives on the device are appropriately sanitized (securely deleted or destroyed) prior to the disposal or repurposing of the system or any storage components.

### Ethics

Ethics approval has been obtained from the NL Health Research Ethics Board (HREB) (20230223). There are no medical risks to participation in this study; however, taking part may make participants feel uncomfortable while discussing their experiences. It will be made clear to participants that they may choose not to answer some of the interview questions or withdraw from the study at any time. Should a participant feel distressed, they will be encouraged by the interviewer to contact any of the organizations listed on the recruitment information and consent form where they will be able to access mental health services confidentially.

## Discussion

The results of this study will help inform the refinement of existing workplace programs and the development of new, practical, and easy-to-implement initiatives to support NGNs’ mental health and well-being as they transition to practice. These initiatives may increase nurse retention, improve patient outcomes, prevent workplace illness, and support nurses who have experienced mental health challenges in their recovery and return to work. By assisting to build a resilient workforce, we hope to reduce workplace-related illnesses associated with poor mental health and wellbeing in future health care crises and positively affect patient care. This research would be of interest to healthcare leadership, policy makers, and nursing organizations interested in promoting the mental health and well-being of RNs, and patient care advocacy groups.

Limitations of this study include potential challenges with participant recruitment and the possibility of NGNs being unable or unwilling to participate due to unavailability of time or hesitancy to recall negative or challenging experiences. Interviews will be conducted by phone or videoconferencing technology to reduce barriers associated with participants’ schedules and eliminate the need for travel. Additionally, the length of interviews have been limited to approximately 60 minutes to minimize the burden of participation in this study. As mentioned, supportive resources will be highlighted in recruitment materials and consent forms to support participants who are distressed by recalling negative or difficult experiences.

Study findings will be disseminated through research reports and presentations with key stakeholders within the province of NL. To reach a wider audience, additional dissemination of findings will take place through journal publications and relevant conference presentations.

## Supporting information

S1 Appendix New Graduate Nurse Interview Guide. (DOCX)
