# Supplementary material for: New graduate nurses’ experiences with and perceptions of their mental health and well-being during the COVID-19 pandemic: An interpretive description study protocol
Source: PLoS One. 2025 Feb 28;20(2):e0315852. doi: 10.1371/journal.pone.0315852 (PMC11870357; doi:10.1371/journal.pone.0315852)
Supplement: S1 Appendix — (DOCX) [file pone.0315852.s001.docx]

**S1 Appendix**

**New Graduate Nurse Interview Guide**

**Demographics**

Age (OR year of birth): __________________

Gender: ________________

Highest Level of Education:

Undergraduate □ Masters □ PhD □

Highest Level of Education in Nursing:

Undergraduate □ Masters □ PhD □

Name of Regional Health Authority where employed: ____________________

Area of practice/specialty: ____________________

Months of experience as a registered nurse: __________

Months of experience in current position: ___________

Position type currently held:

FT □ PT □ Casual □ Permanent □ Temporary □

| **Interview Questions** | |
| --- | --- |
| 1. | Tell me a little about yourself.  *Prompts: Are you from NL? Do you have family or friends close by?* |
| 2. | Tell me about your transition from student nurse to practicing RN  *Prompts: How did it feel? What kind of challenges did you experience? What kind successes did you experience?* |
| 3. | Tell me about your experience starting your first position as a new graduate nurse during COVID-19.  *Prompts: What was it like being oriented to the unit during this time? How did you feel? Did you find it different from your previous clinical experiences before COVID-19?* |
| 4. | How would you describe your mental health and well-being since starting employment as a new graduate nurse?  *Prompt: What do you think influenced/ impacted your mental health the most during this time? Did you have any challenges? Did you have any strategies you used to help in this regard?* |
| 5. | Please describe any supports available to you during this time?  *Prompts: How were you made aware of these supports? Have you ever used any of the supports available to you? If not, please tell us why.*  *Examples of resources could be: Mentors, confidential services, resiliency programs/workshops* |
| 6. | Are there any resources / supports you think would be beneficial in supporting mental health and well-being for new graduate nurses during COVID-19?  *Prompts: Please describe. When would they be offered? By whom? And how? Please describe the process you think would work best.* |
| 7. | Please describe any supports or activities outside of work you use to support your mental health and well-being?  *Prompt: Are these activities helpful? If so, in what way?*  *If asked, examples of resources could be: family and friends, physical activities (walking or running)* |
| 8. | If you could give any advice to a new graduate nurse starting their first position during a public health crisis. What would that be?  *Prompt: If you could go back and tell yourself something you know now that you wish you knew then, what would that be?* |
| 9. | Is there anything else you would like to share or you feel is important for us to know? |
